# c-erbB2 and topoisomerase IIα protein expression independently predict poor survival in primary human breast cancer: a retrospective study

**DOI:** 10.1186/bcr1012

**Published:** 2005-03-21

**Authors:** Peter Fritz, Cristina M Cabrera, Jürgen Dippon, Andreas Gerteis, Wolfgang Simon, Walter E Aulitzky, Heiko van der Kuip

**Affiliations:** 1 Department of Diagnostic Medicine, Pathology, Robert Bosch Hospital, Stuttgart, Germany; 2 Dr Margarete Fischer-Bosch Institute of Clinical Pharmacology, Stuttgart, Germany; 3 Department of Mathematics, University of Stuttgart, Stuttgart, Germany; 4 Department of Gynaecology, Robert Bosch Hospital, Stuttgart, Germany; 5 2nd Department of Internal Medicine, Oncology and Hematology, Robert Bosch Hospital, Stuttgart, Germany

## Abstract

**Introduction:**

c-erbB2 (also known as HER-2/neu) and topoisomerase IIα are frequently overexpressed in breast cancer. The aim of the study was to analyze retrospectively whether the expression of c-erbB2 and topoisomerase IIα protein influences the long-term outcome of patients with primary breast cancer.

**Methods:**

In this study c-erbB2 and topoisomerase IIα protein were evaluated by immunohistochemistry in formalin-fixed paraffin-embedded tissue from 225 samples of primary breast cancer, obtained between 1986 and 1998. The prognostic value of these markers was analyzed.

**Results:**

Of 225 primary breast tumor samples, 78 (34.7%) showed overexpression of either c-erbB2 (9.8%) or topoisomerase IIα protein (24.9%), whereas in 21 tumors (9.3%) both proteins were found to be overexpressed. Patients lacking both c-erbB2 and topoisomerase IIα overexpression had the best long-term survival. Overexpression of either c-erbB2 or topoisomerase IIα was associated with shortened survival, whereas patients overexpressing both c-erbB2 and topoisomerase IIα showed the worst disease outcome (*P *< 0.0001). Treatment with anthracyclines was not capable of reversing the negative prognostic impact of topoisomerase IIα or c-erbB2 overexpression.

**Conclusion:**

The results of this exploratory study suggest that protein expression of c-erbB2 and topoisomerase IIα in primary breast cancer tissues are independent prognostic factors and are not exclusively predictive factors for anthracycline response in patients with primary breast cancer.

## Introduction

The protein expression status of c-erbB2 and, more recently, of topoisomerase IIα has been implicated in the prediction of clinical outcome and response to chemotherapy in breast cancer [1-5]. Gene amplification is the predominant but not exclusive mechanism causing abnormal expression in these tumors (reviewed in [6]). *c-erbB2 *is localized on chromosome 17q12-21 and encodes for a transmembrane tyrosine kinase receptor protein. This highly glycosylated protein is a member of the epidermal growth factor receptor (EGFR; HER) family [7] and is expressed on most cells of epithelial origin. *In vitro*, overexpression of c-erbB2 in epithelial cells ultimately affects the regulation of cell proliferation, of apoptotic pathways, of motility, and of adhesion (overview in [8]). Accordingly, numerous studies have found that both *c-erbB2 *amplification and c-erbB2 protein overexpression predicted disease outcome in patients with localized breast cancer (overview in [9]).

The α isoform of topoisomerase is a key enzyme in DNA replication and also a target for various chemotherapeutic agents such as anthracyclines or epipodophyllotoxins. The gene is located in close proximity to the *c-erbB2 *gene on chromosome 17q21 and encodes for a 170-kilodalton protein. The enzyme catalyzes the unwinding of the DNA to a partly uncoiled form by inducing single-stranded breaks on both DNA strands. These breaks allow the passage of double-stranded DNA through the gap [10]. Anthracyclines, one of the most widely used class of cytotoxic agents for the treatment of breast cancer, inhibit topoisomerase IIα by trapping the DNA strand break intermediates, leading to persistent DNA cleavage.

An important role for the outcome of patients with breast cancer has been proposed both for the c-erbB2 and topoisomerase IIα protein [11]. Interestingly, a high percentage of primary breast tumors with *c-erbB2 *amplification also show a gene copy number alteration of other genes such as *topoisomerase IIα *located near to the *c-erbB2 *locus on chromosome 17 [12,13]. Although the appearance of *topoisomerase IIα *gene alterations (amplification or deletion) was exclusively seen in *c-erbB2*-amplified breast tumors [3,12-14], data on the protein expression status of these two genes in breast tumor samples are controversial. Jarvinen and colleagues report a high correlation of c-erbB2 and topoisomerase IIα protein expression [15]. In contrast, in a recently published study it was observed that in 14 of 33 *topoisomerase IIα*-amplified tumors, topoisomerase IIα protein was present in less than 10% of tumor cells [16]. Another study showed that topoisomerase IIα overexpression is present in 30% of *c-erbB2 *non-amplified tumors [17]. From these studies it has to be concluded that gene amplification is only one of many mechanisms causing high intracellular levels of the topoisomerase IIα protein. This is in concordance with the fact that expression of topoisomerase IIα is regulated on multiple levels, including transcriptional, translational, and post-translational mechanisms [18]. These data suggest that the expression of topoisomerase IIα and c-erbB2 protein might independently predict the clinical outcome in breast cancer. We therefore analyzed the prognostic impact of the protein expression status of both c-erbB2 and topoisomerase IIα in a large cohort of patients with primary breast cancer.

## Materials and methods

### Patients and pathological data

Tumor tissue was analyzed for protein expression of c-erbB2 and topoisomerase IIα from 225 patients with primary invasive breast cancer who underwent surgery from 1986 to 1998 at the Department of Gynaecology, Robert Bosch Hospital, Stuttgart, Germany. The local ethics committee was informed and gave consent. The patient database was anonymized to guarantee privacy. The tissues were formalin-fixed and paraffin-embedded in accordance with standard methods. Histological classification was performed by following the recommendations of the World Health Organization [19]. Three histological types were discriminated: invasive ductal, invasive lobular, and all other specified tumor types such as tubular carcinoma. The pathological reports included tumor size, palpable nodes, metastasis, grading, estrogen receptor status, and progesterone receptor status.

### Immunohistochemical methods

For immunocytochemistry, 3 μm sections were deparaffinized in xylene for 30 min and rinsed in 100%, 96% and 70% ethanol. Sections were then subjected to antigen retrieval by immersion in citrate buffer (pH 6.0) preheated to 99°C for 40 min. Endogenous peroxidase was blocked by incubation in 3% H_2_O_2 _in methanol for 30 min, followed by rinsing in Tris-buffered saline containing Tween 20. Immunohistochemical staining was performed with the EnVision™+ System Kit (DakoCytomation, Glostrup, Denmark). Afterwards, the sections were incubated overnight in a humidity chamber with the monoclonal primary antibody against topoisomerase IIα (DakoCytomation; dilution 1:100) and c-erbB2 (clone CB11; Novocastra, Newcastle, UK; dilution 1:10) followed by incubation for 30 min with a dextran polymer conjugated with horseradish peroxidase enzyme and with goat anti-rabbit antibody. The antigen–antibody immunoreaction was revealed with 3,3'-diaminobenzidinetetrahydrochloride as the chromogen, and the slides were counterstained with hematoxylin.

Immunohistochemical analysis of c-erbB2 (HER-2/neu) protein was also performed with HercepTest™ (DakoCytomation) on an automated immunostaining system (Dako Autostainer), with the use of the manufacture's detection procedures. This procedure was part of the primary diagnostic process. For each case c-erbB2 was assessed twice: once by immunostaining with CB11 and once by herceptin staining. Estrogen and progesterone receptor statuses were assessed during the first years (up to 1990) by charcoal dextran method [20], samples examined after this time were analyzed by immunohistochemistry.

### Scoring interpretation

The scoring system proposed by HercepTest was used for the interpretation of the immunoreactivity of both CB11 and HercepTest, distinguishing between no staining (0), weakly (1+), moderate (2+), and strong membrane staining (3+). Cytoplasmic staining was ignored.

Only nuclear staining was considered for topoisomerase IIα. Immunostaining frequency of the tumor cells was scored subjectively on a scale of 1 to 4 (1, 0 to 5% positive tumor cells; 2, 6 to 25%; 3, 26 to 75%; 4, more than 75%), as proposed by Sandri and colleagues [21]. Finally, after cut-off analysis we stratified the results as negative for less than 25% tumor cells and positive for all cases in which more than 25% of tumor cells stained positive for topoisomerase IIα. For hormone receptor status we classified the tumor as positive if there was more than 15 fmol per mg of protein (charcoal dextran method) or with a score of more than 1 (immunohistochemistry).

### Statistical methods

Descriptive statistical analysis was performed with commercially available software packages (SPSS, version 11.1 (SPSS GmbH, Munich, Germany) and R, version 2.0 ). We used the Kaplan–Meier estimator for univariate statistical analysis and the Cox regression model for multivariate analysis. *P *< 0.05 was considered to be significant. A log-rank test was applied for assessing statistical differences between survival curves. The χ^2 ^test was used to investigate the relationship between topoisomerase IIα expression and histological grading and to analyze the association between topoisomerase IIα and c-erbB2 overexpression with hormone receptor status or stage.

## Results

### Patients

Tissue samples of 225 patients were analyzed for protein expression of c-erbB2 or topoisomerase IIα. The characteristics of these patients and tumors are summarized in Table 1. The distribution of stage, tumor size, nodal status, histological grading, and receptor status is in conformity with that observed in published randomized clinical trials (Table 2; reviewed in [22]). All patients had received appropriate local surgical treatment. Adjuvant medical therapy was given to 143 patients: 61 received hormone therapy; 82 were treated with adjuvant chemotherapy, either anthracycline-containing (46 patients) or anthracycline-free (36 patients; Table 3) regimens. After a mean follow-up period of 67 months, 136 patients were alive and 89 deaths had been recorded. The death of 68 patients (30%) was documented to have been caused by breast cancer (Table 1). Survival at 1 year was 95.73%, at 3 years 85.45%, at 5 years 74.26%, and at 10 years 63.69% for all patients. Again, this survival rate is comparable to that published for similar patient populations [22].

### Prognostic impact of c-erbB2 overexpression

Tumors with an immunoreactive score exceeding 2 or 3 were considered to overexpress c-erbB2. Accordingly, 43 (19.1%) of the patients showed overexpression of this oncogene. Patients with c-erbB2 overexpression showed a similar distribution of age, stage, histological criteria, and receptor status to that of the c-erbB2 negative subgroup (data not shown).

**Figure 1:**
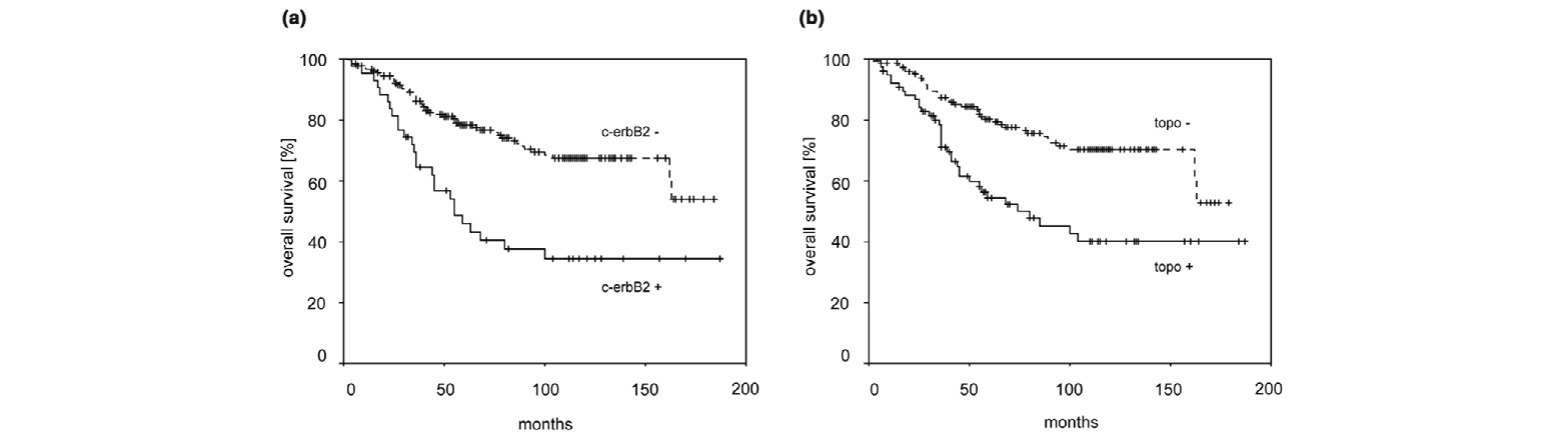
Overall survival of breast cancer patients with regard to c-erbB2 and topoisomerase IIα protein expression. **(a) **Patients with (solid line) or without (dashed line) c-erbB2 protein overexpression. The difference was significant in univariate statistical analysis (log rank 17.94; *P *< 0.0001). **(b) **Significant difference of survival time of patients with tumors positive for topoisomerase IIα protein in more than 25% of tumor cells (solid line) versus patients with tumors with less than 25% topoisomerase IIα-positive cells (dashed line) (log rank 15.59; *P *= 0.0001).

However, the former patients were characterized by a significantly inferior survival in a univariate statistical analysis (log rank 17.94; *P *< 0.0001). The median overall survival time of patients with tumors overexpressing c-erbB2 was 55 months, with a 5-year survival rate of 46.0%. In contrast, median survival time was not reached in the c-erbB2 negative group. The 5-year survival in this group was 78.3% (Fig. 1a). Patients with c-erbB2-overexpressing tumors received no significant benefit from anthracycline-based adjuvant therapy, and even had the worst prognosis of all groups analyzed (log rank 10.17; *P *= 0.001; see below).

### Impact of topoisomerase IIα expression

Topoisomerase IIα staining was strictly nuclear and highly variable between different tissue samples. To study the impact of different levels of overexpression of topoisomerase IIα protein, we analyzed the survival curves of patients with 0 to 5%, 6 to 25%, 26 to 75% and more than 75% positively stained cells (data not shown). These analyses demonstrated that the survival of patients was significantly inferior in cases with high topoisomerase IIα expression in more than 25% of the cells. Therefore, for further analysis overexpression was defined as more than 25% positively stained tumor cells. A subgroup of 77 patients (34.2%) had tumors overexpressing topoisomerase IIα. As shown in Fig. 1b, these patients had a significantly inferior survival (median 80 months; 5-year survival rate 54.4%) in a univariate Kaplan–Meier analysis compared with those without topoisomerase IIα overexpression (median not reached; 5-year survival rate 80.3%; log rank 15.59, *P *= 0.0001). The proportion of tumors expressing topoisomerase IIα increased with histological grading (χ^2 ^test, *P *< 0.001). The fraction of patients with hormone-receptor-positive tumors was significantly higher in samples without topoisomerase IIα overexpression (χ^2 ^test, *P *= 0.004).

**Figure 2:**
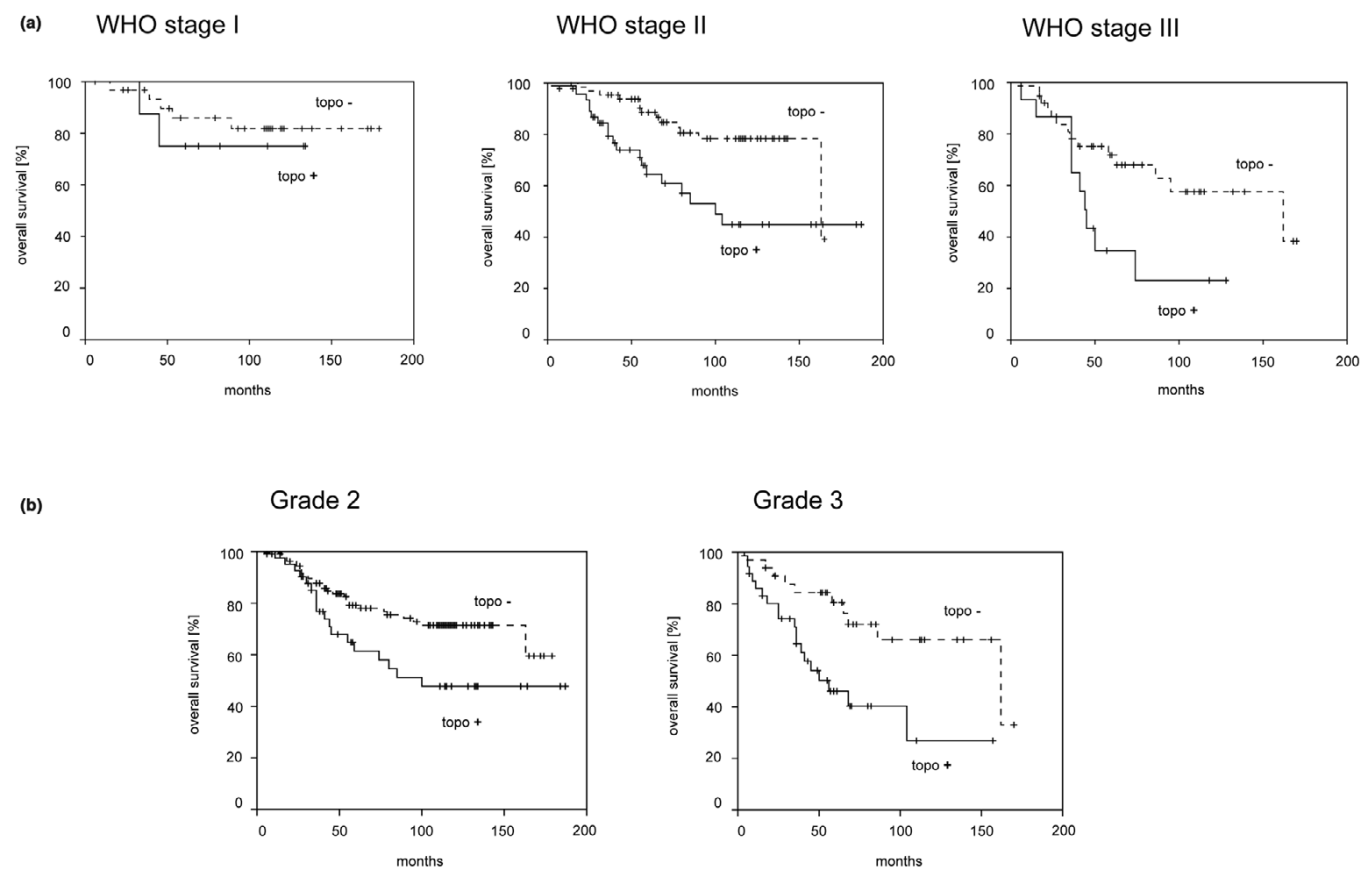
Prognostic impact of topoisomerase IIα protein expression with regard to stage and grading. Patients with tumors positive for topoisomerase IIα in more than 25% of tumor cells (solid lines) were compared with those having tumors expressing topoisomerase IIα in less than 25% of cells (dashed lines) with regard to the stage **(a) **and the grading **(b) **of the tumor. The difference was significant in tumors of stage 2 and 3 (*P *< 0.05) and in grade 2 and 3 tumors (*P *< 0.05).

The prognostic impact of topoisomerase IIα expression was dependent on stage: whereas stage IV patients had an identically poor outcome regardless of topoisomerase IIα expression (not shown), stage II and III patients had a significantly lower survival rate when topoisomerase IIα was highly expressed (log rank 9.35, *P *= 0.002 for WHO stage II; log rank 4.76, *P *= 0.029 for WHO stage III; Fig. 2a). In stage I patients a survival difference of similar magnitude was not statistically significant, probably because of the small sample size (Fig. 2a).

Expression of topoisomerase IIα added prognostic information to histological grading. The analysis was restricted to grade 2 and 3 tumors, because only a few deaths were observed in patients with grade 1 disease. The survival of patients with tumors expressing topoisomerase IIα was inferior in both grade 2 tumors (log rank 5.08; *P *< 0.05) and grade 3 tumors (log rank 7.86; *P *= 0.005; Fig. 2b).

**Figure 3:**
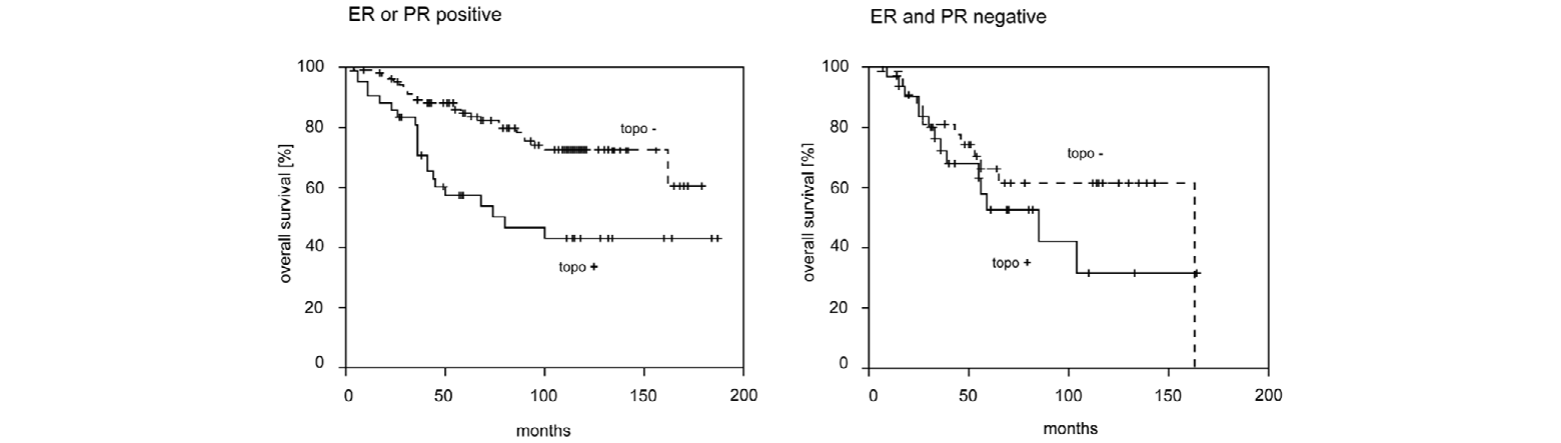
Prognostic impact of topoisomerase IIα protein expression with regard to hormone status. Patients with tumors positive for topoisomerase IIα in more than 25% of tumor cells (solid lines) were compared with those having tumors expressing topoisomerase IIα in less than 25% of cells (dashed lines) with regard to the hormone status. The difference was significant in tumors positive for estrogen receptor or progesterone receptor (log rank 12.0; *P *= 0.0005).

In addition, the prognostic impact of topoisomerase IIα expression was clearly dependent on the steroid hormone receptor status of the tumors: no significant difference was observed in patients with tumors negative for estrogen or progesterone receptor (log rank 0.94; *P *= 0.33; Fig. 3, right panel) whereas detection of topoisomerase IIα in more than 25% of tumor cells identified a subgroup with poor prognosis in receptor-positive breast cancer (log rank 12.0; *P *= 0.0005; Fig. 3, left panel).

**Figure 4:**
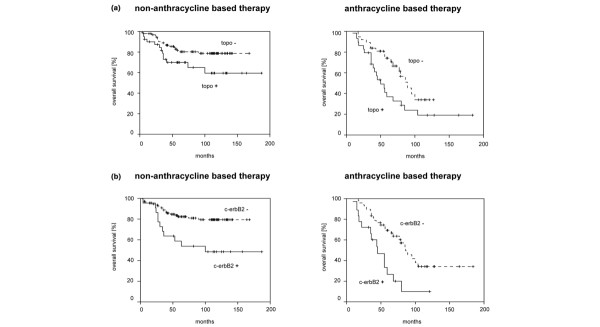
Survival of patients receiving either non-anthracycline-based therapy or anthracycline-based chemotherapy. **(a) **The difference between patients with less than 25% positive cells for topoisomerase IIα (dashed lines) and more than 25% positive cells (solid lines) was significant for both patient groups (*P *< 0.05). **(b) **Significant difference between patients with (solid line) or without (dashed line) c-erbB2 overexpression in the group of patients receiving non-anthracycline-based adjuvant therapy (left panel; *P *= 0.002) or an anthracycline-containing regimen (right panel; *P *= 0.001)

We further studied whether the prognostic impact of topoisomerase IIα expression was restricted to patients receiving either non-anthracycline-containing or anthracycline-containing regimens for adjuvant treatment. By analogy with the results obtained with c-erbB2-overexpressing tumors (Fig. 4b), treatment with anthracyclines was not capable of reversing the negative prognostic impact of topoisomerase IIα expression (log rank 4.74; *P *= 0.02; Fig. 4a).

### Coexpression of c-erbB2 and topoisomerase IIα

**Figure 5:**
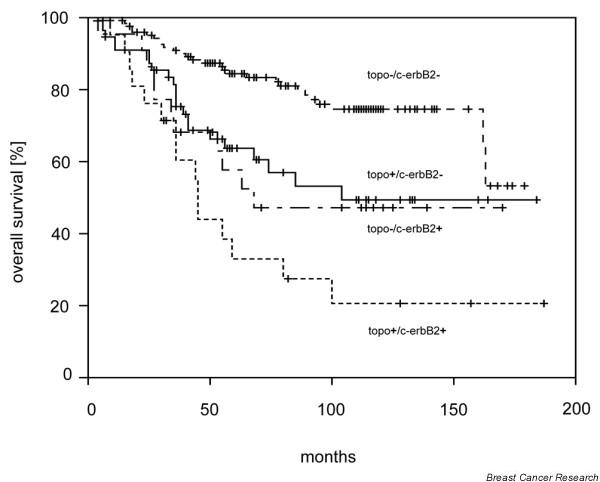
Survival of patients with and without overexpression of c-erbB2 and topoisomerase IIα. The difference between patients without overexpression of c-erbB2 or topoisomerase (broken line), patients with tumors overexpressing one of the two proteins (c-erbB2, dot–dashed line; topoisomerase IIα, solid line), and patients with tumors overexpressing both c-erbB2 and topoisomerase IIα (dotted line) was significant (log rank 29.71; *P *< 0.0001).

The subgroups overexpressing c-erbB2 and topoisomerase IIα were overlapping but not identical. One hundred and twenty-six tumors (56%) showed neither c-erbB2 nor topoisomerase IIα overexpression. Fifty-six (25%) patients had tumors overexpressing topoisomerase IIα only; 22 (9.8%) showed only c-erbB2 overexpression. Twenty-one (9.3%) tumors showed increased staining for both proteins. None of these groups differed significantly with regard to clinical stage (Table 4). However, a statistically significantly higher proportion of hormone-receptor-negative cancers was observed in the group overexpressing one or both proteins studied (χ^2 ^test, *P *= 0.006; Table 4). A survival analysis of these four groups demonstrated an independent negative prognostic impact of either overexpression. As shown in Fig. 5, patients with breast cancer lacking overexpression of both c-erbB2 and topoisomerase IIα had the best long-term prognosis (the median survival time was not reached; the 5-year survival rate was 84.4%). Overexpression of either c-erbB2 or topoisomerase IIα was associated with intermediate survival (for c-erbB2-overexpressing tumors the median survival time was 68 months and the 5-year survival rate was 57.7%; for topoisomerase IIα-overexpressing cases the median survival time was 104 months and the 5-year survival rate was 63.7%). Those patients overexpressing both proteins had the worst outcome, with a median survival of 45 months and a 5-year survival rate of 33.0% (log rank 29.71; *P *< 0.0001; Fig. 5).

In a multivariate Cox regression model (Tables 5 and 6), tumor stage, estrogen receptor, topoisomerase IIα, and c-erbB2 all independently predicted disease-related death. Subsequent inclusion of grading was far beyond statistical significance (Table 5). Adding the interaction term topoisomerase IIα by grading (topoisomerase IIα × grading; Table 5) seems worth consideration. However, in a Wald statistics grading together with the last-mentioned interaction term failed to reach significance (*P *= 0.11). Models containing in addition interactions of c-erbB2 by grading (c-erbB2 × grading; Table 5) and c-erbB2 by topoisomerase IIα (c-erbB2 × topoisomerase IIα; Table 5) are not significantly superior. The last two interaction terms are therefore not included in the final model as presented in Table 6.

## Discussion

Overexpression of c-erbB2 and topoisomerase IIα independently predicts poor survival in this retrospective series of patients. Topoisomerase IIα and c-erbB2 were found to be overexpressed in overlapping but distinct subgroups of patients. Moreover, the prognostic impact of topoisomerase IIα overexpression seems to be independent of other prognostic variables in a multivariate analysis and makes the prognosis significantly worse in both c-erbB2-positive and c-erbB2-negative patients. In addition, the prognostic impact of topoisomerase IIα overexpression is dependent on the steroid receptor status. The results of this study suggest that anthracycline treatment is not capable of reversing the negative prognostic influence of topoisomerase IIα or c-erbB2 expression.

Recent reports have studied the interaction between topoisomerase IIα expression and pathological variables, chemotherapy response, and the proliferation rate of breast cancer. However, only very limited series reported a long-term outcome of patients with primary breast cancer depending on topoisomerase IIα. Di Leo and colleagues [2] studied patients participating in a randomized clinical trial comparing CMF (cyclophosphamide, methotrexate, and 5-flourouracil) with an anthracycline-containing regimen. The subgroup of patients with *topoisomerase IIα *amplification did not do obviously more badly than the remaining patients. However, the outcome was strongly dependent on the adjuvant therapy applied. Another series measuring topoisomerase IIα protein by immunohistochemistry showed a significantly adverse influence of topoisomerase IIα expression of a similar magnitude to that in our study [23]. Durbecq and colleagues [16] have shown that measurement of protein expression and gene amplification each identify different subsets of patients. These data suggest that detection of the protein might be better correlated with the biological properties of the tumor and therefore might predict the clinical outcome more precisely than genetic analysis. The data in our study support the view that protein expression of topoisomerase IIα is a relevant factor predicting long-term prognosis in patients with newly diagnosed breast cancer independent of c-erbB2.

Treatment of cell lines with anthracyclines was less effective in cells with a low expression of topoisomerase IIα [24,25]. A retrospective subgroup analysis of a randomized clinical trial suggests that the prognostic impact of *topoisomerase IIα *gene amplification is restricted to the patients not receiving anthracycline-based chemotherapy [2]. Furthermore, other studies that related tumor response to anthracyclines also showed some correlation between gene amplification status of *topoisomerase IIα *and clinical response [3-5]. This is in contrast with a recently published retrospective study in which amplification of *c-erbB2 *and *topoisomerase IIα *was not predictive of the response to anthracycline [26]. Our analyses show that the negative prognostic impact of topoisomerase IIα protein overexpression is observed both in patients who received an anthracycline-containing regimen for adjuvant chemotherapy and in those who did not. Moreover, the difference in survival between topoisomerase IIα-positive and topoisomerase IIα-negative patients exceeds the proportion of patients expected to be cured by chemotherapy. Protein overexpression has been related to a variety of other molecular markers predictive for high proliferation rate or high grading of malignancy such as Ki67 expression or aneuploidy [16,23,26,27]. These findings support the view that overexpression of topoisomerase IIα protein indicates a poor prognosis irrespective of the therapy applied. *Topoisomerase IIα *gene amplification is much more closely associated with *c-erbB2 *amplification than the protein expression status (overview in [6]). The predictive value of the gene amplification for clinical and long-term outcome might therefore be related to amplification of either or both of the *c-erbB2 *or *topoisomerase IIα *genes. As coamplification is the predominant mechanism of genetic alteration of these two genes in breast cancer, clinical observations separate the role of each gene in the clinical response to adjuvant treatment with anthracyclines.

The biological role of topoisomerase IIα overexpression is unknown. Cells lacking topoisomerases II are not capable of finishing a normal cell cycle and should therefore not be viable [28,29]. In addition, it has been shown that experimental overexpression of topoisomerase IIα in different human cell lines causes apoptosis [30]. From these observations one must conclude that cells staining with a low intensity are not cells with a complete lack of topoisomerase IIα function. This is in conformity with the observation that normal cells are also weakly positive for topoisomerase IIα. High expression might be related to a high proliferation rate. This view is supported by our finding that topoisomerase IIα expression is related to histological grading and by observations showing some correlation of topoisomerase IIα with Ki67 expression and other proliferation markers [23,27]. However, viable cells with constitutive overexpression might also indirectly indicate defects in apoptotic pathways. Both hypotheses might provide a plausible explanation for the observation that topoisomerase IIα overexpression is related to a more aggressive tumor phenotype.

## Conclusion

Our data suggest that protein expression of topoisomerase IIα is a prognostic factor that is independent of c-erbB2, stage, and histological grading. In addition, the results of this exploratory study indicate that anthracycline treatment is not capable of reversing the negative prognostic influence of the expression of topoisomerase IIα or c-erbB2. Nevertheless, because of the small number of patients remaining in these subgroups, no firm conclusion can be made about the predictive value of topoisomerase IIα or c-erbB2 regarding sensitivity to anthracyclines. Our results support the view that studying the deregulation of either the *topoisomerase IIα *gene or topoisomerase IIα protein might yield different results depending on the method applied. This should be considered when planning prospective studies on the predictive and prognostic value of topoisomerase IIα.

## Competing interests

The author(s) declare that they have no competing interests.

## Authors' contributions

PF, WS, WEA, JD, and HvdK were responsible for generating the hypothesis and correcting the manuscript. PF, AG, and WS were responsible for surgery and for collecting the patient material. PF, CMC, and HvdK were responsible for immunostaining and for examination and interpretation of the results. PF, JD, and WEA performed the statistical analysis of the data. PF, HvdK, and WEA were responsible for writing the manuscript. All authors read and approved the final manuscript.

## Figures and Tables

**Table 1:** Characteristics of patients and tumors

Characteristic	Value
Number of patients	225
Age, years (median (range))	56 (29–88)
Time to relapse, months (median (range))^a^	25 (0–179)
Alive without disease	123
Relapse	23
Disease status unknown	3
Alive (censored)	136
Death	89
By tumor	68
By unknown causes	8
Not tumor-related (censored)	13

^a^Only cases with recurrence are included in this calculation.

**Table 2:** Clinical characteristics of study patients (*N *= 225)

Variable	*n *(%)
Stage (*n *= 214)	
I	40 (18.7)
II	113 (52.8)
III	53 (24.8)
IV	8 (3.7)
T (*n *= 222)	
T1	59 (26.6)
T2	106 (47.7)
T3	27 (12.2)
T4	30 (13.5)
N (*n *= 222)	
N0	94 (42.3)
N1	112 (50.5)
N2-3	16 (7.2)
Grading (*n *= 220)	
G1/G2	151 (68.6)
G3	69 (31.4)
Histology (*n *= 225)	
Ductal	175 (77.8)
Lobular	22 (9.8)
All other subtypes	28 (12.4)
Menopausal (*n *= 163)	
Premenopausal	43 (26.4)
Postmenopausal	120 (73.6)
ER (*n *= 212)	
Positive	135 (63.7)
Negative	77 (36.3)
PR (*n *= 211)	
Positive	127 (60.2)
Negative	84 (39.8)
Metastasis (*n *= 218)	
Negative	210 (96.3)
Positive	8 (3.7)

ER, estrogen receptor; N, palpable nodes; PR, progesterone receptor; T, tumor size.

**Table 3:** Medical treatment for primary breast cancer in the study patients (*N *= 198)

Therapy type	Intervention	*n *(%)
Adjuvant hormone therapy	No hormone therapy	137 (69.2)
	Tamoxifen	61 (30.8)
Adjuvant chemotherapy	Anthracycline-containing regimens	46 (23.2)
	Anthracycline-free regimens	36 (18.2)
	No chemotherapy	116 (58.6)
	No adjuvant therapy	55 (27.8)

**Table 4:** Clinical characteristics of patients with and without overexpression of c-erbB2 and topoisomerase IIα

Parameter	Value			
	
Topoisomerase overexpression	Absent	Absent	Present	Present
c-erb B2 overexpression	Absent	Present	Absent	Present
Stage, *n *(%)				
I	29 (24.2)	3 (15.0)	6 (11.1)	2 (10.0)
II	59 (49.1)	7 (35.0)	36 (66.6)	11 (55.0)
III	29 (24.2)	9 (45.0)	9 (16.7)	6 (30.0)
IV	3 (2.5)	1 (5)	3 (5.6)	1 (5.0)
Total^a^	120	20	54	20
Receptor status, *n *(%)				
ER or PR positive	92 (79.3)	12 (57.1)	32 (58.2)	10 (52.6)
PR and PR negative	24 (20.7)	9 (42.9)	23 (41.8)	9 (47.4)
Total^b^	116	21	55	19

^a^214 patients with complete data were analyzed for stage; ^b^211 patients with complete data were analyzed for receptor status. ER, estrogen receptor; PR, progesterone receptor.

**Table 5:** Choice of the Cox regression model (*N *= 196)

Stepwise inclusion of variable	2 × increase in partial log-likelihood (χ^2 ^distributed)	df	*P*
Stage	23.43	3	< 0.0001
Estrogen receptor	7.92	1	0.005
c-erbB2	7.34	1	0.01
Topoisomerase IIα	9.85	1	0.002
Grading	0.01	1	0.92
Topoisomerase IIα × grading	4.64	1	0.03
c-erbB2 × grading	1.74	1	0.19
c-erbB2 × topoisomerase IIα	0.17	1	0.68

Starting from the null model, successive adding of the variables mentioned above improves the Cox proportional hazard model in the first four steps significantly. In this sense the first four variables can be considered as independent prognostic factors. df, degrees of freedom.

**Table 6:** Coefficients of the linear predictor in the final Cox regression model (*N *= 196)

Variable	*B*	exp(*B*)	*P*	95% CI for exp(*B*)
Stage				
II^a^	0.72	2.06	0.14	0.79–5.36
III^a^	1.69	5.4	0.001	1.97–14.74
IV^a^	3.25	25.68	<0.001	6.72–98.17
Estrogen receptor (pos. versus neg.)	0.58	1.79	0.039	1.03–3.11
c-erbB2 (pos. versus neg.)	0.82	2.26	0.0036	1.31–3.93
Topoisomerase IIα (pos. versus neg.)	0.39	1.48	0.005	0.77–2.83
Grading (3 versus {1,2})	t0.62	0.54	0.15	0.23–1.25
Topoisomerase IIα × grading^b^	1.21	3.35	0.035	1.09–10.36

^a^The hazard ratio exp(*B*) of the stage given is the relative risk of this stage with respect to stage I. ^b^The effect of the interaction term on survival can be described as follows: if grading equals G1 or G2, the hazard ratio of topoisomerase IIα positive versus negative is 1.48 (95% confidence interval (CI) 0.77 to 2.83); if grading equals G3, this hazard ratio is 4.96 (95% CI 2.00 to 12.29).
